# Stabilizing Sleep–Wake Cycles and Social Functioning in Bipolar Disorders: Effect of Interpersonal and Social Rhythm Therapy

**DOI:** 10.3390/jcm15031071

**Published:** 2026-01-29

**Authors:** Mona Metwally El-Sayed, Dauda Salihu, Abdelaziz Hendy, Loujain Sharif, Khalid Sharif

**Affiliations:** 1 Department of Community, Psychiatric, and Mental Health Nursing, College of Nursing, Qassim University, Buraydah 52571, Saudi Arabia; m.mona@qu.edu.sa (M.M.E.-S.); d.salihu@qu.edu.sa (D.S.); 2 Department of Maternal and Child Health Nursing, College of Nursing, Qassim University, Buraydah 52571, Saudi Arabia; 3 Psychiatric and Mental Health Nursing Department, Faculty of Nursing, King Abdulaziz University, Jeddah 21589, Saudi Arabia; lsharif@kau.edu.sa; 4 Department of Behavioral Medicine and Psychiatry, West Virginia University, Morgantown, WV 26505, USA; khsharif@hsc.wvu.edu

**Keywords:** patient care, interpersonal and social rhythm therapy, circadian rhythm stability, social functioning, bipolar disorders

## Abstract

**Background**: Functional impairments associated with bipolar disorder have a significant impact on daily life, including work, social relationships, and independent living. Bipolar disorder is treated with many approaches, with pharmacotherapy being the first choice; however, cases of relapse and side effects have been reported. The literature suggests that psychosocial interventions are effective in improving treatment adherence, recognizing early warning signs, enhancing self-management skills, and fostering open communication. The effects of interpersonal and social rhythm therapy (IPSRT) on circadian rhythm stability and social functioning in people with bipolar disorder remain uncertain. Therefore, this study is needed. **Methods**: This quasi-experimental study was conducted in the psychiatric outpatient clinic of a university hospital. Participants were recruited using convenience sampling from the psychiatric outpatient clinic. Eligible participants were then randomly allocated to either the intervention or control group using a coin-flip method. The dose of the intervention averaged 75 min per session with a weekly frequency over 12 weeks. Outcome measures included the Interpersonal Problem Areas Rating Scale, the Social Rhythm Metric Scale-II-5, and the Multnomah Community Ability Scale. Data were collected at baseline (week 0), post-intervention (week 12), and at follow-up (12 weeks post-intervention), and analyzed using repeated-measures ANOVA. **Results**: Participants in the IPSRT group demonstrated significant improvements in social rhythm regularity (SRM-II-5: 2.9 ± 1.3 at baseline, 3.7 ± 1.2 post-intervention, and 4.0 ± 1.5 at three-month follow-up; F = 18.5, *p* < 0.05, η^2^ = 0.37). A significant between-group difference favoring IPSRT emerged at three months (t = 3.01, *p* < 0.05, d = 0.76). Social functioning also improved significantly in the intervention group (MCAS: 55.5 ± 7.4 at baseline, 63.7 ± 7.1 post-intervention, and 62.3 ± 6.9 at follow-up; F = 29.4, *p* < 0.05, η^2^ = 0.49). Between-group differences were significant immediately post-intervention (t = 4.10, *p* < 0.001, d = 1.05) and at three-month follow-up (t = 2.73, *p* = 0.008, d = 0.72). **Conclusions**: IPSRT produced sustained improvements in social rhythm stability and social functioning, demonstrating its clinical value in the management of bipolar disorder.

## 1. Introduction

Since Kraepelin’s early distinction between manic–depressive illness and schizophrenia, bipolar disorder (BD) has historically been viewed as having a relatively favorable prognosis, based on the assumption that it is less commonly associated with progressive deterioration and functional impairment [1,2]. Bipolar disorder is widespread, and many people experience an alternation between manic and depressive states. Within the spectrum of bipolar disorder, a wide range of clinical symptoms can develop, but all are persistent, disabling, and recurrent. Mania, hypomania, mixed states, and depression are common symptoms of bipolar disorder, often occurring in late childhood or early adolescence [3]. In many cases, bipolar disorder is not diagnosed until early adulthood, even though symptoms have been present for years. Although bipolar disorder is characterized by periods of relative health interrupted by episodes of illness, most people with bipolar disorder are sad on a daily basis. However, manic and hypomanic symptoms characterize bipolar disorder [4]. With an estimated lifetime prevalence of 1.5% to 6%, bipolar spectrum disorder is a significant cause for concern [5].

The National Survey on the Prevalence of Mental Disorders in Egypt surveyed 14,640 adults aged 18 to 64 years in five regions and found that 6.43% of the population suffered from a mental disorder, including bipolar disorder [6]. Although early research placed limited emphasis on the psychosocial consequences of bipolar disorder (BD), growing evidence highlights substantial and persistent functional impairment associated with the condition [7]. Recent studies have demonstrated that significant psychosocial dysfunction may persist even during euthymic periods, indicating that symptom remission does not necessarily translate into functional recovery [8]. For example, a longitudinal study of hospitalized patients experiencing a first manic episode with psychotic features found that while most individuals achieved symptomatic recovery within a 24-month follow-up, only a small proportion attained functional recovery, underscoring a marked disparity between clinical improvement and social functioning in BD [9]. Poor functional outcomes in BD have been associated with multiple factors, including comorbid substance use, medication side effects, a history of psychotic symptoms, low premorbid functioning, persistent subsyndromal mood fluctuations, a greater number of previous episodes and hospitalizations, earlier age at onset, and ongoing cognitive impairment [10]. Collectively, these impairments substantially reduce health-related quality of life and limit individuals’ capacity to maintain employment and productivity [5,11].

Pharmacotherapy is generally the first choice in the treatment of manic, depressive, and residual states in individuals with bipolar disorder; however, cases of relapse and side effects have been reported [12]. It is worth noting that the relapse rate of patients after psychosocial therapy has been shown to decrease [13,14]. This could be due to the success of psychosocial treatment in improving treatment adherence, recognizing early warning signs, improving self-management skills, and promoting open communication within the family [13,14].

A number of non-pharmacological interventions for bipolar disorder focus on lifestyle changes, psychological therapies, and support systems to manage symptoms and maintain mood stability [6]. These include psychotherapy (e.g., cognitive behavioral therapy (CBT) [15], interpersonal and social rhythm therapy (IPSRT) [16], and family-focused therapy (FFT) [14].

These psychosocial interventions have been studied and have been shown to be successful in improving treatment adherence, recognizing early warning signs, enhancing self-management skills, and promoting open communication [14,17]. However, the effects of interpersonal and social rhythm therapy (IPSRT) on circadian rhythm stability and social functioning in individuals with bipolar disorder are unclear. This study, therefore, aims to explore this important niche.

Interpersonal and Social Rhythm Therapy (IPSRT), a psychotherapy based on the theory of circadian zeitgebers in affective disorders, assumes that regular external events such as social interactions, daily routines, and environmental stimuli can synchronize the internal biological clock with ecological rhythms [18]. These external cues, or zeitgebers, can influence or disrupt the internal circadian rhythm, thereby affecting mood and overall well-being. The theory is that disruptions to these zeitgebers can trigger mood swings in individuals prone to mood disorders [13,19]. IPSRT aims to regulate the sleep–wake cycle and circadian rhythm by addressing interpersonal issues and modifying the circadian rhythm [20,21].

IPSRT was developed with careful consideration of potential triggers, making it a practical approach to managing chronic diseases [22]. With this method, individuals are guided and trained on how to maintain a consistent daily routine while being given a safe space to express their feelings of sadness and anger about the illness [23]. By recognizing that the disease will be a lifelong challenge, IPSRT might help to reduce denial and improve treatment adherence. Once a routine is established, therapists work with patients to anticipate and manage potential disruptions to their rhythm, such as home visits or vacations [24]. IPSRT aims to enhance the stability of these rhythms and help patients become aware of how to manage potential disruptions [25,26].

## 2. Method

### 2.1. Research Design

This study used a quasi-experimental research design with repeated measurements, which complies with TREND (Transparent Reporting of Evaluations with Nonrandomized Designs) guidelines [27]. The study was conducted over approximately six months, from March to August 2024, including a 12-week intervention phase followed by a 12-week follow-up period.

### 2.2. Setting

The study was conducted in the outpatient clinics at Al-Maamoura Psychiatric Hospital in Alexandria, affiliated with the General Secretariat of Mental Health. This hospital provides complementary treatment for patients with psychiatric disorders. These clinics offer a range of services, including assessments, diagnoses, and medication prescriptions. They are open from 8 a.m. to 1 p.m. on a specific day each week. The research was conducted in a comfortable room within the outpatient clinic on a designated working day to maintain privacy and confidentiality during sessions.

### 2.3. Ethical Considerations

The study was conducted in accordance with the Declaration of Helsinki and approved by the Institutional Review Board of the College of Nursing at Alexandria University (IRB00015625/7/1/2024), approval date 1 July 2024 and the Human Rights Protection Committee of the General Secretariat of Mental Health, Ministry of Health and Population in Cairo (code 989/2024), approval date 2 March 2024. Informed consent was obtained from all subjects involved in the study.

### 2.4. Participants

Participants were recruited using convenience sampling from the psychiatric outpatient clinic. Eligible participants were then randomly allocated to either the intervention or control group using a coin-flip method.

To ensure eligibility, participants’ medical records were meticulously reviewed. They were included if they (1) had a diagnosis of bipolar disorder I or II as defined by the Diagnostic and Statistical Manual of Mental Disorders, Fifth Edition (DSM-5) [28], (2) were ≥18 years old, (3) attended the psychiatric outpatient clinic, (4) had no comorbidities, (5) were able to read, write, and communicate in English and Arabic, and (6) consented to participate in the study. The exclusion criteria include individuals who are in an acute phase of their illness or who exhibit psychotic traits as documented in their medical records. At baseline, participants were clinically assessed by the treating psychiatrist and were required to be in a non-acute phase of illness. Individuals experiencing acute manic, depressive, or psychotic episodes at the time of recruitment were excluded.

### 2.5. Interventions

Interpersonal and Social Rhythm Therapy (IPSRT) is a structured psychotherapy intervention designed to enhance mood and functioning in individuals with bipolar disorder. The lead researcher, who is a professor of psychiatric and mental health nursing with formal training in psychotherapeutic interventions, delivered the Interpersonal and Social Rhythm Therapy (IPSRT). Prior to study commencement, the researcher completed an online IPSRT certification program (IPSRT.org), comprising approximately 20 h of curriculum on theory, clinical structure, and application. Throughout the intervention period, the researcher participated in biweekly supervision with an IPSRT-certified clinician to review cases, discuss treatment fidelity, and address protocol-related challenges. Adherence to the IPSRT manual was monitored using structured session logs that documented key intervention components across each phase, such as social rhythm monitoring, interpersonal problem-solving, and psychoeducation. The clinical supervisor reviewed a randomly selected subset of session records to assess consistency with the IPSRT framework. While formal fidelity ratings were not conducted, supervision notes and session documentation were used to verify adherence to the intended protocol.

The intervention group received 12 IPSRT sessions, spread over three months, during which clients met individually with the trained researcher once a week for an average of 75 min per session. This schedule is tailored to the client’s ability to complete the sessions. IPSRT was originally developed by Klerman [29] for the treatment of unipolar depression, and was later modified by Frank et al. [22] specifically for bipolar disorder. The therapy focuses on stabilizing the participants’ everyday routines and improving the quality of their interpersonal relationships and social role fulfillment. With this approach, IPSRT aims to enhance participants’ current mood and level of functioning while equipping them with the necessary skills to prevent future episodes of affective distress. Therapy is divided into three phases: an initial phase, an intermediate phase, and a continuation/maintenance phase, with the frequency of sessions decreasing over time as the client progresses (Table 1).

The researchers followed a structured process to recruit individuals with bipolar disorder for the IPSRT intervention after carefully reviewing the medical records of potential participants to ensure that they met the inclusion criteria. Each participant was interviewed individually by the principal investigator for 30–45 min in a quiet, private room. During these interviews, the researcher established rapport and explained the purpose of the study, the goals of IPSRT, the number and length of sessions, and the expected weekly homework assignments. The researcher also obtained informed consent from participants and collected their contact information to facilitate follow-up and plan future appointments. The lead researcher then created a written schedule for the IPSRT sessions, scheduling appointments based on the availability of both the participant and the researcher. Between sessions, the lead researcher offered participants telephone counseling twice a week. Throughout the intervention, he documented participants’ responses, feedback on the most valuable skills, estimated time, and any obstacles they encountered.

All participants continued their prescribed pharmacological treatment as usual throughout the study period. No protocol-driven medication changes were introduced by the research team. According to medical records and participant reports, no major medication changes occurred during the intervention and follow-up period.

#### Control Group

A control group continued to receive their usual care (TAU), including the use of one or more mood stabilizers and atypical antipsychotics, as recommended by their psychiatrist in the outpatient clinic. Assessments and evaluations were conducted before the intervention group began IPSRT sessions and three months later, at the end of the study.

### 2.6. Hypotheses

**H1.**  *Individuals with bipolar disorder attending IPSRT sessions would show greater circadian rhythm stability than the control group*.

**H2.**  *Individuals with bipolar disorder who participate in IPSRT sessions would exhibit higher levels of social behavior than the control group*.

### 2.7. Outcomes

Demographic data (i.e., gender, age, education, occupation, marital status, religion, income level, region of residence, type of housing, and support system). Clinical data includes information such as duration of illness, number of previous hospitalizations, type of last episode, current medications prescribed, and medication adherence. The primary outcomes were (1) interpersonal relationship problems (i.e., interpersonal deficits, 2) interpersonal role conflict, unresolved grief, loss of healthy self, and role switching, and (2) circadian rhythm and social functioning. Trained research assistants collected data at baseline (T0), post-intervention (T1) after 12 weeks, and at follow-up (T2) after three months, using the same assessment tools, MCAS and SRM-II-5. Additionally, they contacted participants by phone and through WhatsApp group messages to facilitate follow-up assessments.

The researcher utilized demographic and clinical data, as well as the MCAS, SRM-II-5, and IPARS-M, to identify any interpersonal issues or social role changes that might be associated with the client’s emotional episodes and social routines. The IPARS-M was administered once during the initial assessment phase to determine each participant’s specific interpersonal problem areas. This guided the focus of the subsequent intervention sessions. Upon completing this phase, the researcher identified specific interpersonal problem areas, including unresolved grief, role transitions, loss of a healthy self, role conflicts, and broader interpersonal challenges.

#### 2.7.1. Measures

##### Demographic and Clinical Data Sheet for Individuals Diagnosed with BD

The questionnaire was designed to collect information about individuals diagnosed with bipolar disorder (BD). It included questions on age, gender, marital status, education level, region of residence, monthly income, presence of a support system (family members or friends), and housing situation. Additionally, the sheet elicited clinical information or illness histories, such as the type of the patient’s most recent episode, the duration of their illness, the number of previous hospital admissions, the prescribed medications, and medication compliance.

##### Social Rhythm Metric Scale-II-5 (SRM-II-5)

The SRM-II-5, a shortened version of the original Social Rhythm Metric (SRM) developed by Monk [30], is a reliable instrument for measuring daily circadian rhythms. It focuses on five important daily activities: waking up, first contact with another person, the start of work (including paid work, volunteer work, housework, childcare, or school), dinner time, and bedtime. The SRM-II-5 records the times of these activities and calculates regularity by averaging the number of times each activity is performed within 45 min of the usual time over seven days. The scores range from 0 to 7, indicating the regularity of the daily routine. In a study by Monk et al. [31], the scale’s sensitivity was reported to be 74%, its specificity 95%, and its applicability 0.7, demonstrating its effectiveness in measuring daily social rhythms. The scale also showed good internal consistency, with a Cronbach’s alpha of 0.87, further emphasizing its reliability.

##### Interpersonal Problem Area Rating Scale-Modified (IPARS-M)

The Interpersonal Problem Area Rating Scale (IPARS) was originally developed by Klerman [29] within the framework of interpersonal psychotherapy to identify key interpersonal problem areas associated with mood disorders. In the present study, a modified version of the scale (IPARS-M), adapted from the version described by Fontes de Andrade et al. [32], was employed. The IPARS-M assesses five interpersonal problem areas, including interpersonal deficits, interpersonal role conflicts, unresolved grief, role transitions, and loss of a healthy self. Participants were asked to identify the interpersonal problem area that best characterized their current difficulties, and each area was rated according to its perceived importance using a three-point ordinal scale (1 = most significant problem, 2 = secondary problem, 3 = less significant problem). The scale was administered once at baseline to characterize participants’ interpersonal difficulties and to guide the focus of the IPSRT intervention. An example item from the IPARS-M is: “Which of the following interpersonal areas best describes the main difficulty you are currently experiencing?” The IPARS-M was translated into Arabic using a forward–backward translation procedure. The Arabic version demonstrated good psychometric properties in the current study, with high internal consistency (Cronbach’s α = 0.88). Factor analysis supported the construct validity of the scale, with factor loadings exceeding acceptable thresholds and Bartlett’s test of sphericity indicating suitability for factor analysis (*p* < 0.001).

##### Multnomah Community Ability Scale (MCAS)

The MCAS is a standardized instrument for assessing the social functioning and abilities of individuals with psychiatric disorders. It assesses various aspects of independent functioning [33]. Each item is scored on a five-point scale, with lower scores indicating poorer functioning. The items are divided into four areas: Interference with Functioning (5 items measuring psychiatric symptoms, physical health, and intellectual function), Adjustment to Living (3 items evaluating daily living skills and acceptance of illness), Social Competence (5 items assessing social interest, skills, and meaningful activity), and Behavioral Problems (4 items measuring treatment participation, substance use, and acting-out behaviors). The items in the first three areas are assessed for the last three months, while the fourth area covers the last year. The total score ranges from 17 to 85, with scores between 17 and 47 indicating severe impairment, scores between 48 and 62 indicating moderate impairment, and scores between 63 and 85 indicating mild impairment. With a Cronbach’s alpha value of 0.84, it has good internal consistency [34]. The researchers carefully translated and back-translated the scale. Factor loadings above the accepted threshold of 0.35 explained 89.58% of the total variance, with values ranging from 0.83 to 0.94 after rotation. The Kaiser–Meyer–Olkin value for sampling adequacy was an impressive 0.96, and Bartlett’s test for sphericity yielded a statistically significant result (*p* < 0.001), confirming the appropriateness of the scale items. Additionally, the scale demonstrated high reliability, with a Cronbach’s alpha value of 0.87.

### 2.8. Sample Size Calculation

The required sample size was calculated using G*Power 3.1.9.7 [35], based on an effect size of 0.8, α = 0.05, and 85% power to detect differences between the two groups at three measurement time points: T0 (baseline), T1 (post-intervention), and T2 (follow-up). This calculation resulted in a target sample size of 30 participants per group (60 in total). Considering a possible dropout rate of 10% (n = 6), a total of 66 subjects is required (33 in each of the intervention and control groups).

### 2.9. Assignment Methods

Although this study employed a quasi-experimental design, random assignment was implemented to enhance group comparability. A coin flip was used to allocate participants to either the IPSRT group or the treatment-as-usual control group. The randomization sequence was generated and applied by the lead researcher [36].

### 2.10. Blinding

Given the nature of the intervention, participants and the therapists delivering IPSRT were aware of group assignments. However, to minimize bias in outcome assessment, the research assistants who collected post-intervention and follow-up data were blinded to group allocation. This was achieved by keeping assessment records separate from intervention logs and ensuring that outcome assessors were not involved in treatment delivery or session scheduling. Additionally, data analysts were blinded to group labels until all statistical analyses were completed.

### 2.11. Unit of Analysis

This study’s unit of analysis included individuals diagnosed with bipolar disorder. Analysis was conducted at the group level, and statistical comparisons, including repeated-measures ANOVA and independent *t*-tests, were performed to examine differences between the intervention and control groups at time points T0, T1, and T2.

### 2.12. Statistical Methods

The data were analyzed using IBM SPSS Statistics version 26.0 (IBM Corp., Armonk, NY, USA) [37]. The clinical profile and demographic data of the participants were described based on the frequency and percentage according to their level of measurement. Normality was tested using the Kolmogorov–Smirnov (KS) and Shapiro–Wilk (SW) tests. The normality of continuous variables was assessed using the Shapiro–Wilk test, and all distributions did not significantly deviate from normality (*p* > 0.05), supporting the use of parametric statistical tests.

Changes in groups at different times within each group were investigated through repeated measures analysis of variance (ANOVA) on each group separately (intervention and control groups), with time (pretest, posttest, and follow-up) as the within-group variable. If sphericity was present, tests were evaluated with standard F values; otherwise, the Greenhouse-Geisser correction method was employed. Effect sizes of the repeated measures ANOVA test were calculated through partial eta squared (η^2^), where values of 0.01, 0.06, and 0.14 stood for a small, moderate, and large effect, respectively.

Differences between groups at all time points were examined with independent samples *t*-tests. Welch’s *t*-test was utilized when there was a violation of homogeneity of variance assumptions. Effect sizes from between-group contrasts were determined using Cohen’s d, with 95% confidence intervals included to assess the accuracy of effect size estimates. Small, medium, or large effect sizes were delineated by Cohen’s d values of 0.20, 0.50, or 0.80, respectively. All statistical tests were two-tailed, and a *p* value of less than 0.05 was considered statistically significant.

### 2.13. Pilot Investigation

Ten subjects with bipolar disorder were used in a preliminary study to assess the clarity of the instruments (MCAS, IPARS-M) and procedures and to investigate any barriers to intervention implementation and data collection. The preliminary investigation has shown that the research instruments are clear, practicable, and easy to understand. The study was conducted smoothly and without any obstacles. It is worth noting that the subjects involved in the preliminary study were not included in the main study.

## 3. Results

### 3.1. Participant Flow

**Figure 1:**
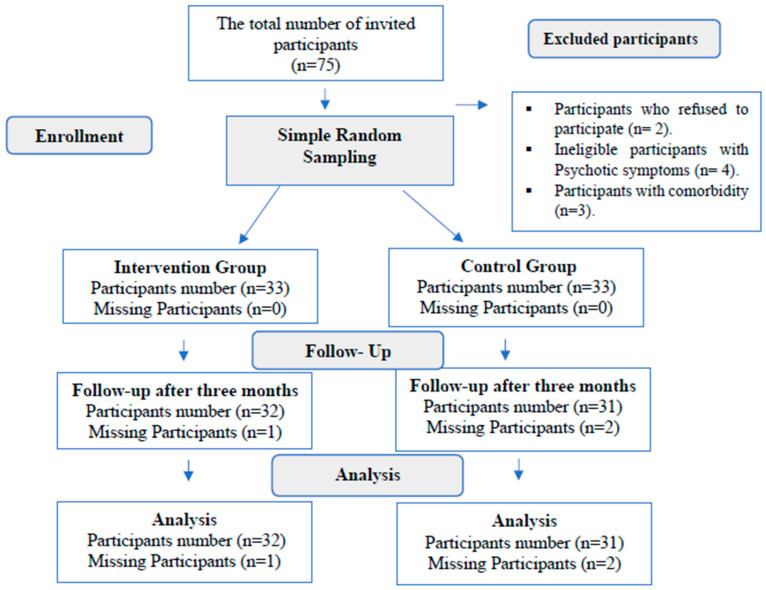
Participants’ Recruitment Flowchart.

Initially, 75 patients were invited to participate in the study. However, 4 participants were deemed ineligible due to their medical records indicating they were in an acute phase with psychotic symptoms such as delusions, severe flight of ideas, distractibility, and pressure of speech. Additionally, 3 participants had a comorbid substance use disorder (SUD) with bipolar disorder (BPD). Furthermore, 2 patients refused to participate in the study. However, after completion of the IPRST sessions, one patient in the intervention group and two patients in the control group did not complete the follow-up test. Therefore, the final number of participants in the intervention group who completed all the intervention sessions and the follow-up was 32, while in the control group, there were 31 participants (Figure 1).

### 3.2. The Participants’ Characteristics

#### 3.2.1. Demographics

As shown in Table 2, the two groups were similar in terms of age, with the majority being between 20 and 29 years old (49% in the intervention group and 45.2% in the control group). Males constitute the majority (40.7% in the intervention and 35.5% in the control groups). The marital status was evenly distributed between the two groups. The level of education was also similar, with most participants having a secondary or university degree. In terms of occupation, most participants in both groups were unemployed (59.4% in the study group and 64.5% in the control group). Monthly income was also comparable, with around half of the participants in each group having a reasonably sufficient income. The region of residence and living conditions were also similar in both groups, with most participants living in urban areas and with their families.

#### 3.2.2. Clinical Data

Illness duration was comparable between the two groups, with most participants having a disease duration of less than five years (56.3% in the intervention group and 61.3% in the control group). The number of previous hospitalizations was also similar, with most participants having either no or one previous hospitalization. The nature of the final episode was also comparable, with most participants in both groups experiencing either depression or mania. The medications currently prescribed were also similar, with most participants in both groups taking mood stabilizers, either alone or in combination with antipsychotics. Medication compliance and the presence of a support system were also comparable in both groups. Overall, the demographic and clinical characteristics of the study group and the control group matched well, with no statistically significant differences between the two groups. At baseline, both groups were clinically stable and comparable in terms of demographic and clinical characteristics.

**Interpersonal Relationship Problem Areas**

Table 3 shows that the most common difficulty in both groups was interpersonal deficiencies, with 34.4% in the intervention group and 32.3% in the control group. The second most common area was interpersonal role conflicts, which affected 28.1% of the intervention group and 25.8% of the control group. The other problem areas, such as unresolved grief, loss of healthy self, and role transitions, were less common in both groups. Unresolved grief was reported by 12.5% of the intervention group and 16.1% of the control group. Loss of healthy self was reported by 15.6% of the intervention group and 19.4% of the control group. Role transitions were reported by 9.4% of the intervention group and 6.5% of the control group. The distribution of IPARS-M difficulty domains was not statistically significant between the intervention and control groups, as indicated by the chi-square test (χ^2^ = 1.902, *p* = 0.661). This might indicate that the two groups were well matched in terms of the interpersonal difficulties they experienced.

**Circadian Rhythm**

Table 4 presents the mean score of SRM-II-5 of participants in the intervention and control groups at all three assessment points. Within-group analysis shows that the SRM-II-5 scores resulted in a statistically significant change in the intervention group over time, F = 18.5, *p* < 0.05, partial η^2^ = 0.37 (large). On the other hand, no statistically significant change appeared in the control group, F = 2.10, *p* > 0.05, with a small effect size, partial η^2^ = 0.07.

Comparisons between the groups revealed no significant differences at baseline or immediately after intervention, while a statistically significant difference favoring the intervention group was detected three months after the study intervention commencement. The t-value is 3.01 at *p* < 0.05, with a large effect size, Cohen’s d = 0.76, 95% CI: 0.25–1.27. This would imply that improvements in social rhythm regularity through IPSRT were not only statistically but also clinically significant and maintained over time.

**Social Functioning**

Table 5 presents the mean changes in MCAS scores for participants in the intervention and control groups across the three measurement points. Within-group comparison indicated that the MCAS score had a statistically significant improvement in the intervention group across time (F = 29.4, *p* < 0.05). The partial η^2^ had a large effect size (0.49), indicative of a strong and clinically meaningful effect by the application of IPSRT. In contrast, no statistically significant change was detected in the control group (F = 2.40, *p* > 0.05), although the corresponding effect size was small (partial η^2^ = 0.07).

Between-groups comparison showed that there was no statistically significant difference in MCAS scores at baseline, with *p* > 0.05, thus confirming the baseline equivalence between the two groups. Significantly higher MCAS scores were observed in the intervention group immediately after intervention: t = 4.10, *p* < 0.001, with a large effect size, Cohen’s d = 1.05, 95% CI: 0.54–1.56. This significant difference remained evident at three-month follow-up, t = 2.73, *p* = 0.008, with a moderate-to-large effect size, Cohen’s d = 0.72, 95% CI: 0.20–1.23.

## 4. Discussion

To our knowledge, this is the first study to comprehensively examine the effects of Interpersonal and Social Rhythm Therapy on circadian rhythms and their broader effect on various aspects of social functioning and interpersonal relationship problems (i.e., interpersonal deficits, interpersonal role conflict, unresolved grief, loss of healthy self, and role transitions).

Interpersonal and Social Rhythm Therapy (IPSRT) has been shown to significantly enhance circadian rhythm regulation and improve overall social functioning in individuals diagnosed with bipolar disorder. This therapeutic approach has also been effective in reducing various forms of impaired social functioning, such as interference with daily functioning, difficulties in adapting to life changes, challenges in social competence, and behavioral issues. Furthermore, IPSRT has addressed several interpersonal relationship difficulties, including interpersonal deficits, conflicts in social roles, unresolved grief, the loss of a healthy sense of self, and difficulties in role transitions.

Circadian rhythm and social function in bipolar disorder have been shown to be affected by both mood state and pharmacological therapy. Even though participants had a stable level of psychopathology at baseline and received treatment as usual, a lack of standardized assessment of mood severity and pharmacological treatment makes it difficult to distinguish the specific effect of IPSRT independent of these factors. Thus, results concerning improved circadian rhythm and social function must be taken in a proper predictive frame.

The observed improvements can be attributed to the core mechanisms of IPSRT, the stabilization of circadian rhythms, and the strengthening of adaptive coping strategies. As indicated by the literature, IPSRT’s structured approach to regulating daily routines and resolving interpersonal conflicts appears to modulate important biological and psychosocial processes in bipolar disorder [6,38]. By reinforcing consistent social and behavioural zeitgebers, IPSRT can help synchronize circadian gene expression [39]. Additionally, the reliance on self-report measures of sleep–wake cycles, factors known to influence mood stability [23,40]. These findings are consistent with those of Alam et al. [24], who reported that bipolar patients who received IPSRT showed significant improvements in symptom severity, sleep disturbance, and psychological adjustment compared to control subjects. Similarly, ref. [19] found that IPSRT in combination with pharmacotherapy led to greater reductions in affective and anxiety symptoms as well as better social functioning. Long-term benefits were also demonstrated in a 12-month study by Frank [22], in which concomitant IPSRT led to lower relapse rates for both manic/hypomanic and depressive episodes compared to drug treatment alone.

During the 12-week IPSRT, significant improvements in MCAS social skills were noted immediately after the intervention and three months later. These results may be related to the planned regular social interactions and support from friends and family, which appear to have a protective effect against bipolar episodes. Spending time with loved ones on a regular basis can help to strengthen social functioning and increase feelings of support [19,40]. It is important to recognize that Frank [22] emphasized that working with bipolar patients to overcome interpersonal challenges can lead to more social support, less stress, better emotional processing, and improved interpersonal skills.

One of the key findings of this study was that IPSRT significantly improved adjustment to the living environment, a crucial aspect of community integration. For example, the ability to manage finances, care for oneself, and run a household improved. This may be related to the preliminary findings linking IPSRT to increased prefrontal cortical activity and neurotrophic factor levels. These neurobiological changes could strengthen the neural circuits that support self-regulation and goal-directed behavior [16].

The study indicated that a considerable number of participants exhibited significant interpersonal deficits, particularly in the development and maintenance of relationships. This is observed in the high proportion of single and unemployed participants, which suggests significant professional and interpersonal impairments. Similarly, previous research has consistently shown that interpersonal dysfunction is prevalent in individuals with bipolar disorder, even during periods of euthymia when overt symptoms are minimal [19,41]. Difficulties in developing and maintaining healthy relationships can lead to loneliness, isolation, loss of social support, and increased life stress [10,42].

In addition to the significant interpersonal deficits identified, the study revealed that a considerable number of the participants suffered from interpersonal role conflicts and interpersonal deficiencies. This was often reflected in a high rate of marital problems, including separation and divorce, among the participants. Evidently, previous research has consistently documented that separation and divorce rates are two to three times higher in individuals with bipolar disorder compared to the general population [12,43,44]. This might be related to role ambiguity, excessive demands, and authority conflicts that often occur in these relationships. The mood swings associated with bipolar disorder can easily lead to anger and conflict, further straining the marital relationship [12]. In addition, the cognitive distortions that often occur during bipolar episodes, such as magnification, generalization, and dichotomous thinking, can have a detrimental effect on communication patterns and overall relationship dynamics [40]. This interplay of interpersonal, cognitive, and mood-related factors likely contributes to the high rates of marital problems observed in this population [2].

This study also found that many of the participants reported struggling with unresolved grief. These individuals were struggling with the complicated and unresolved experience of losing a loved one, which they were unable to process and cope with adequately. The results of the current study are consistent with the broader literature and confirm that unresolved grief reactions are common in individuals with bipolar disorder. These unresolved grief experiences appear to contribute to the broader impairments in functioning observed in this clinical population [14,26,43].

The current study found that many participants experienced profound grief following their bipolar disorder diagnosis, often describing a sense of loss of a healthy self. This grief appeared to exacerbate feelings of stigma and was compounded by a limited understanding of the disorder and its management. These findings aligned with the qualitative work of Proudfoot [45], who examined the experiences of 26 individuals with recently diagnosed bipolar disorder. Their participants similarly reported a disrupted sense of self, fears about an uncertain future, and stigma as dominant concerns post-diagnosis. Future studies should incorporate standardized mood rating scales and longitudinal medication monitoring, and consider mixed-effects modeling to better isolate intervention-specific effects.

### 4.1. Limitations of the Study

This study has several limitations that should be considered when interpreting the findings. First, the sample was relatively small and recruited from a single university-based outpatient clinic, which may limit the generalizability of the results to broader or more diverse clinical populations. Second, reliance on self-report measures introduces the potential for reporting bias. Third, the three-month follow-up period may not capture the long-term sustainability of treatment effects. Fourth, the absence of qualitative data limits deeper insight into participants’ personal experiences of the intervention. Most notably, the study lacked an attention-matched control condition. The intervention group received substantially more therapeutic contact, weekly individual sessions, and phone follow-ups than the treatment-as-usual control group. Therefore, observed improvements may reflect non-specific factors such as increased clinician attention, therapeutic support, or participant expectancy, rather than the specific mechanisms of IPSRT. Future research should address these limitations by employing larger, more diverse samples, incorporating objective and behavioral measures, extending follow-up duration, integrating qualitative methods, and critically including an active control condition (e.g., supportive therapy with equivalent contact time) to isolate the unique contribution of IPSRT beyond general therapeutic effects. This study did not include standardized longitudinal assessments of manic or depressive symptom severity.

### 4.2. Conclusion and Recommendations

The results of this study suggest that the IPSRT intervention has led to an improvement in circadian rhythm, life adjustment, and social functioning in people with bipolar disorder. It was also found to reduce impaired social functioning, interpersonal conflict, loss of healthy self, and role transition. These findings emphasize the importance of incorporating IPSRT interventions into treatment plans for individuals with bipolar disorders to improve their overall well-being and quality of life.

### 4.3. Relevance for Clinical Practice

The results of this study have important implications for nursing practice in the care of people with bipolar disorder. Nurses play a crucial role in implementing and delivering IPSRT interventions, which significantly improve the stability of the circadian rhythm and social functioning. Incorporating IPSRT into their practice can help patients establish and maintain a regular daily routine, maintain interpersonal relationships, and participate more effectively in social activities. Providing psychoeducation to patients and their families promotes a better understanding of the disorder and the importance of adherence to IPSRT intervention.

## Figures and Tables

**Table 1:** IPSRT Session Schedule.

Phases	Tasks
Initial phase (3–5 sessions)	This initial intervention depends on the length and complexity of the client’s emotional history, interpersonal interactions, and the amount of psychoeducation necessary.
Intermediate phase (3 sessions)	- This phase of the intervention focused on assisting the client in developing more regular daily social routines and resolving the interpersonal issues identified in the earlier phase of treatment. Psychoeducation was provided on the necessity of social rhythm stabilization and how to apply the SRM-II-5. Participants were required to complete the SRM-II-5 daily. - During the sessions, the client’s SRM-II-5 from the previous week was reviewed, and events and actions that disrupted social rhythms and how they affected his mood were discussed.
Continuation or maintenance phase (4 sessions)	- This phase focused on instilling confidence in clients’ abilities to apply the techniques learned during the acute phase of therapy to preserve their present euthymic mood, level of functioning, and social rhythm regularity. The goal was for the client to maintain regular social routines despite pressures such as work changes, vacations, and other unexpected life events. - The client was urged to continue to increase the quality of their interpersonal interactions while reducing the amount of interpersonal distress. - Techniques for accomplishing these interpersonal goals include “Communication Analysis,” which allows the client and researcher to identify problem areas and help the client interact more effectively with significant others. - “Role-playing” provides a safe environment for the client to practice expressing emotions and self-assertion. - “Decision Analysis” assists clients in reflecting on the potential risks and benefits of choices and options for a specific problem.
Final phase (2 sessions)	The last phase of IPSRT included efforts to end therapy or reduce the frequency of sessions even further. It also involved an assessment of the client’s treatment achievements and areas of susceptibility for future episodes (Frank et al. [22])

**Table 2:** Demographic and clinical characteristics of the participants.

	Intervention Group (n = 32)		Control Group (n = 31)		Test of Sig.	*p*
**Demographic Data**	No.	%	No.	%	***χ***^2^ = 0.270	^MC^*p* = 0.703
**Age**						
20–29	15	49.0	14	45.2		
30–40	7	21.0	6	19.4		
>40	10	30.0	11	35.4		
**Gender**					***χ***^2^ = 0.021	0.871
Male	13	40.7	11	35.5		
Female	19	59.3	20	64.5		
**Marital Status**					***χ***^2^ = 0.062	^MC^*p* = 1.000
Single	14	43.8	15	48.4		
Married	12	37.5	11	35.5		
Divorced	6	18.7	5	16.1		
**Education levels**						
Primary	0	0.0	1	3.2	***χ***^2^ = 2.100	0.147
Secondary	13	40.6	14	45.2		
University	19	59.4	16	51.6		
**Occupation**						
Employed	13	40.6	11	35.5	***χ***^2^ = 0.045	0.729
Unemployed	19	59.4	20	64.5		
**Monthly Income**						
Sufficient	10	31.3	11	35.5	***χ***^2^ = 1.706	0.279
Sufficient to some extent	13	40.6	12	38.7		
Insufficient	9	28.1	8	25.8		
**Residence Region**						
Urban	20	62.5	21	67.7	***χ***^2^ = 1.271	^MC^*p* = 0.894
Rural	12	37.5	10	32.3		
**Living Arrangements**						
With family	24	75.0	25	80.6		
Alone	8	25.0	6	19.4	***χ***^2^ = 0.065	0.799
**Support System**						
Yes	18	56.3	19	61.3		
No	14	43.7	12	38.7	***χ***^2^ = 1.150	0.347
**Clinical Data**						
**Duration of Illness**						
<5 years	18	56.3	19	61.3		
≥5 years	14	43.7	12	38.7	***χ***^2^ = 0.241	0.426
**Number of Previous Hospital Admissions**						
None	12	37.5	10	32.3		
One	8	25.0	9	29.0	***χ***^2^ = 0.121	0.962
Two	7	21.9	8	25.8		
≥Three	5	15.6	4	12.9		
**Type of Last Episode**						
Depression	14	43.8	16	51.6		
Mania	15	46.8	13	41.9	***χ***^2^ = 3.450	^MC^*p* = 0.919
Hypomania	3	9.4	2	6.4		
**Medications currently prescribed**						
Mood Stabilizers	17	53.1	18	58.0		
Antipsychotics	8	25.0	7	25.8	***χ***^2^ = 1.860	0.449
Both	7	21.9	6	19.2		
**Medications Compliance**						
Compliance	24	75.0	22	71.0		
Noncompliance	8	25.0	9	29.0	***χ***^2^ = 0.780	^MC^*p* = 0.957

χ^2^: Chi-square test; MC: Monte Carlo; *p*: *p*-value for comparing the two studied groups. Statistically significant at *p* ≤ 0.05.

**Table 3:** Distribution of the participants according to the IPARS-M.

	Intervention Group (n = 32)		Control Group (n = 31)		Test of Sig.	*p*
IPARS-M	No.	%	No.	%	1.902	^MC^*p* = 0.661
Interpersonal Deficiencies	11	34.4	10	32.3		
Interpersonal Role Conflicts	9	28.1	8	25.8		
Unresolved Grief	4	12.5	5	16.1		
Loss of Healthy Self	5	15.6	6	19.4		
Role Transitions	3	9.4	2	6.5		

IPARS-M: Interpersonal Problem Area Rating Scale- Modified; MC: Monte Carlo; *p*: *p*-value for comparing the two studied groups. Statistically significant at *p* ≤ 0.05.

**Table 4:** Mean scores of the participants on the SRM-II-5 before, immediately after, and three months after the application of IPSRT.

Group/Time Point	Pretest M (SD)	Immediate Post M (SD)	3-Month Follow-Up M (SD)	F (Within Group) *p* Value	Partial η^2^
Intervention (n = 32)	2.9 (1.3)	3.7 (1.2)	4.0 (1.5)	18.5 <0.05	0.37
Control (n = 31)	2.3 (1.7)	3.0 (1.9)	2.9 (1.4)	2.10 >0.05	0.07
*t* test *p* value	1.57 >0.05	1.68 >0.05	3.01 <0.05		
Cohen’s d 95% CI for d	0.40 −0.09 to 0.89	0.44 −0.05 to 0.93	0.76 0.25 to 1.27		

Values are presented as mean (SD). Within-group effects were examined using repeated-measures ANOVA. Between-group differences at each time point were examined using independent samples *t*-tests (Welch). Effect sizes are reported as partial eta squared (η^2^) for ANOVA and Cohen’s d with 95% confidence intervals for between-group comparisons. SRM-II-5: Social Rhythm Metric Scale-II-5.

**Table 5:** Mean scores of the participants on the MCAS before, immediately after, and three months after applying IPSRT.

Group/Time Point	Pretest M (SD)	Immediately Post M (SD)	3-Month Follow-Up M (SD)	F (Within Group)	*p* Value	Partial η^2^
Intervention (n = 32)	55.5 (7.4)	63.7 (7.1)	62.3 (6.9)	29.4	<0.05	0.49
Control (n = 31)	53.8 (9.9)	55.3 (8.9)	56.0 (10.3)	2.40	>0.05	0.07
*t* test *p* value	0.79 >0.05	4.10 <0.001	2.73 0.008			
Cohen’s d 95% CI for d	0.19 −0.30 to 0.68	1.05 0.54 to 1.56	0.72 0.20 to 1.23			

Values are presented as mean (SD). Within-group effects were examined using repeated-measures ANOVA. Between-group differences at each time point were examined using independent samples *t*-tests (Welch). Effect sizes are reported as partial eta squared (η^2^) for ANOVA and Cohen’s d with 95% confidence intervals for between-group comparisons.

## Data Availability

The data sets used or analyzed in this study are available on request from the corresponding author.
